# Commentary: Usage of Mitogen-Activated Protein Kinase Small Molecule Inhibitors: More Than Just Inhibition!

**DOI:** 10.3389/fphar.2018.00935

**Published:** 2018-08-20

**Authors:** Marius Pollet, Jean Krutmann, Thomas Haarmann-Stemmann

**Affiliations:** ^1^IUF-Leibniz Research Institute for Environmental Medicine, Düsseldorf, Germany; ^2^Medical Faculty, Heinrich-Heine University, Düsseldorf, Germany

**Keywords:** aryl hydrocarbon receptor, kinase inhibitor, off-target effects, signal transduction, pregnane X receptor

Steffen Meurer and Ralf Weiskirchen recently published an interesting and important study concerning the off-target effects of so-called “specific” protein kinase inhibitors (PKI) that are frequently applied in both, basic research and clinical applications (Meurer and Weiskirchen, 2018). A PKI-dependent inhibition of non-targeted protein kinases may occur due to the usage of concentrations that exceed the respective IC_50_ value by multiple factors. Meurer and Weiskirchen instead describe a PKI-mediated activation of non-targeted protein kinases. Specifically, the authors observed that a treatment of hepatic stellate cells, hepatocytes and portal myofibroblasts with a chemical inhibitor for a certain mitogen-activated protein kinase (MAPK) led to an activation of other members of the MAPK network. The authors called this phenomenon “activation by inhibition” and “cross-activation” (Meurer and Weiskirchen, 2018), terms which well describe the experimental observations but not the underlying molecular mechanism.

Regarding the latter one, we realized that four of the five MAPK inhibitors tested in the respective study are known to interfere with the activity of the aryl hydrocarbon receptor (AHR), a ligand-activated transcription factor and key regulator of xenobiotic metabolism (Murray et al., 2014). In its inactive form, the AHR is trapped in a cytosolic multiprotein complex. Upon binding of small molecular weight compounds, this complex dissociates and the AHR shuttles in the nucleus, dimerizes with its partner molecule ARNT and induces gene expression (Murray et al., 2014). The probably best-examined AHR target genes encode for the xenobiotic-metabolizing monooxygenases cytochrome P450 (CYP) 1A1, CYP1A2, and CYP1B1, which, in most cases, oxidize the invading chemicals to enhance their polarity and facilitate their excretion (Mescher and Haarmann-Stemmann, 2018). Importantly, the ligand-driven activation of AHR is frequently accompanied by a stimulation of other cellular signaling pathways, including NF-κB, epidermal growth factor receptor (EGFR) and MAPK signal transduction (Haarmann-Stemmann et al., 2009; Puga et al., 2009; Tian, 2009). The list of AHR ligands encompasses infamous environmental pollutants, such as 2,3,7,8-tetrachlorodibenzo-*p*-dioxin (TCDD) and benzo[*a*]pyrene, plant polyphenols, microbiota-derived indoles and phenazines as well as several pharmaceuticals (Murray et al., 2014). Interestingly, more than a dozen PKI, including the four MAPK inhibitors SB203580, U0126, PD98059, and SP600125 tested by Meurer and Weiskirchen, have been identified to date to modulate AHR activity and downstream gene expression (e.g., of CYP1A1) in either a positive or a negative manner (Table 1). Several PKI, such as the phosphoinositide 3-kinase inhibitor LY294002, bind to the AHR protein and antagonize its activation by the prototype ligand TCDD (Guo et al., 2000), whereas others, including the aforementioned U0126 and SB203580, were shown to interact with AHR and increase its transcriptional activity (Andrieux et al., 2004; Korashy et al., 2011). In this context, it is interesting to know that an activation of AHR by different ligands has been reported to stimulate the phosphorylation of ERK1/2, p38 MAPK, JNK, and upstream receptor tyrosine kinases in various human and rodent cells (Haarmann-Stemmann et al., 2009; Puga et al., 2009). In fact, Fumio Matsumura and his team have been among the first reporting a direct impact of AHR activation on protein kinase activity. Specifically, they observed an increased activity of protein kinase C and EGFR in hepatic tissue of rodents treated with TCDD (Madhukar et al., 1984; Bombick et al., 1985). Further examples for an impact of AHR activation on the signal transduction network, are the TCDD-induced phosphorylation of p38 MAPK observed in hepatoma cells (Weiss et al., 2005) and macrophages (Park et al., 2005), as well as the activation of EGFR and downstream ERK1/2 signaling by the AHR agonists 6-formylindolo[3,2-*b*]carbazole and TCDD in keratinocytes and colon cancer cells, respectively (Fritsche et al., 2007; Xie et al., 2012). It is thus tempting to speculate that at least some of the PKI-induced off-target effects observed by Meurer and Weiskirchen, such as the phosphorylation of ERK1/2 and JNK by the p38 MAPK inhibitor SB203580 or the activation of JNK and p38 MAPK by the MEK1/2 blocker U0126, were due to a stimulation of AHR activity. One may describe this phenomenon as an effect of a certain PKI on a non-kinase target (Munoz, 2017) or simply as the recognition of a foreign compound by the cellular defense system against xenobiotics. This notion is supported by the fact that some PKI have been found to interact with other xenobiotic receptors as well. The MEK1/2 inhibitor U0126, for instance, was shown to induce the expression of CYP3A4 in human hepatoma cells by binding to the pregnane X receptor (PXR) (Smutny et al., 2014). In addition, five out of nine tested clinically relevant PKI (erlotinib, gefitinib, nilotinib, sorafenib, and vandetanib) induced the expression of the ATP-binding cassette transporter P-glycoprotein in a PXR-dependent manner in human colon cancer cells (Harmsen et al., 2013).

**Table 1:** Overview on protein kinase inhibitors known to manipulate AHR signaling.

**Inhibitor**	**Target kinase**	**IC_50_** **target kinase [μM]^a^**	**AHR** **modulation**	**Tested cells/cell-lines/tissue**	**IC_50_/EC_50_ AHR-specific endpoint [μM]**	**References**
AG-494	EGFR	1	Inhibitor^b^	Human Caco-2 colon cancer cells	Not available	Kasai and Kikuchi, 2010
Akti-1/2	AKT1 AKT2	0.05 0.21	Inhibitor^c^	Human MCF-7 breast cancer cells	IC_50_: 5.86	Gilot et al., 2010
LY294002	PI3Kα PI3Kβ PI3Kδ	0.5 0.97 0.57	Antagonist	Human MCF-10A mammary epithelial cells	IC_50_: 35	Guo et al., 2000
PD98059	MEK	2	Antagonist	MCF-10A cells	IC_50_: 1–4	Reiners et al., 1998
PP2	SFK Fyn SFK Hck SFK Lck SFK Src	0.005 0.005 0.004 0.1	Agonist	Human HepG2 hepatoma cells, human NCTC 2544 keratinocytes	Not available	Frauenstein et al., 2015
SB203580	p38	0.0003–0.0005	Agonist	Murine Hepa1c1c7 hepatoma cells, HepG2	Not available	Korashy et al., 2011
SB216763	GSK3	0.034	Partial agonist	Hepa1c1c7 cells, murine PW531 hepatoma cells, murine primary hepatocytes	Not available	Braeuning and Buchmann, 2009
SP600125	JNK1/2 JNK3	0.04 0.09	Antagonist/partial agonist	Hepa1c1c7 cells, HepG2 cells, rat liver, human primary hepatocytes	IC_50_: 1.5–7 EC_50:_ 0.005–1.89	Joiakim et al., 2003 Dvorak et al., 2008
STO-609	CaMKKα/β	0.027	Agonist	MCF-7 cells, human primary macrophages, human A549 lung cancer cells	EC_50:_ 0.043–3.4	Monteiro et al., 2008
SU11248	c-Kit CSF1R FGFR1 FLT3 PDGFRα PDGFRβ RET VEGFR1/2	0.001–0.01 0.05–0.1 0.88 0.25 0.069 0.039 0.05 0.004	Inducer^c^	MCF-7 cells	Not available	Maayah et al., 2013
TSU-16/SU5416	VEGFR2	1.2	Agonist	HepG2 cells, human primary hepatocytes, human 101L hepatoma cells, rat 5L hepatoma cells, murine primary splenocytes	EC_50_: 0.007–9.8	Mezrich et al., 2012 Matsuoka-Kawano et al., 2010
TSU-68/SU6668	AURKB AURKC FGFR1 PDGFRβ VEGFR2	0.035 0.21 3 0.06 2.43	Inducer^b^	Human primary hepatocytes, rat liver	Not available	Kitamura et al., 2008a Kitamura et al., 2008b
U0126	MEK1 MEK2	0.07 0.06	Agonist	Human B16A2 hepatoma cells, rat primary hepatocytes	EC_50:_ 2.5	Andrieux et al., 2004

a *IC_50_ values for target kinase(s) and respective references are provided by Cayman Chemical (www.caymanchem.com) and Selleckchem (www.selleckchem.com), respectively. The IC_50_ values for SU11248 are from Heng and Kollmannsberger (2010)*.

b *Mode of action not clear*.

c *Ligand-independent mode of action*.

*AURKB, aurora kinase B; AURKC, aurora kinase C; AKT, protein kinase B; CaMKK, calcium/calmodulin-dependent protein kinase kinase; CSF1R, colony stimulating factor 1 receptor; c-kit, mast/stem cell growth factor receptor; EGFR, epidermal growth factor receptor; FGFR, fibroblast growth factor receptor; FLT3, Fms-like tyrosine kinase 3; GSK3, glycogen synthase kinase 3; JNK, c-Jun N-terminal kinase; MEK, mitogen-activated protein kinase kinase; PI3K, phosphoinositide-3-kinase; RET, rearranged during transfection; SFK, Src family kinases; p38 MAPK, p38 mitogen-activated protein kinase; PDGFR, platelet-derived growth factor receptor; VEGFR, vascular endothelial growth factor receptor*.

The majority of PKI interact with the ATP-binding cleft of the target enzyme (Bain et al., 2007; Wu et al., 2015). Given that the 3D structure of the ATP-binding cleft is highly conserved amongst eukaryotic protein kinases, these inhibitors are limited in both their size and structural diversity (Bain et al., 2007; Wu et al., 2015). As implied by the growing list of PKI identified to interact with AHR and PXR, the structural prerequisites to bind to the ATP-binding cleft of protein kinases seem to resemble those required to interact with the ligand-binding domain of the xenobiotic receptors. Indeed, AHR and PXR contain a relatively large ligand-binding domain with a cavity volume of ~840 Å^3^ (Denison et al., 2002) and ~1,150 Å^3^ (Watkins et al., 2001), respectively, and share an extreme structural diversity of ligands (Denison and Faber, 2017). Notably, the known IC_50_/EC_50_ values of some PKI to modulate AHR activity are indeed in the range of the IC_50_ for their target kinases (Table 1). However, apart from ligand-binding, certain PKI may indirectly interfere with AHR signaling, for instance by inhibiting protein kinases contributing to AHR's nuclear translocation (Haarmann-Stemmann et al., 2009).

We agree with the authors that a detailed knowledge of the off-target effects induced by widely used PKI is urgently required, not only for the proper interpretation of experimental data, but in particular, to better forecast potential drug-drug interactions during therapy. When working with so-called “specific” PKI, one should be aware that these compounds will be at first recognized by the chemical defense system, i.e. by xenobiotic receptors, drug-metabolizing enzymes and drug transporters, of the exposed cells. The interaction with one or more xenobiotic receptor(s) is determined by the structural and physiochemical properties of a given PKI and may not only affect its own metabolism, but also the metabolism of eventually co-administered drugs, and the activity of other signaling pathways that might be tightly interconnected with the addressed chemosensory receptor.

## Author contributions

MP performed literature research and created the table. JK revised the manuscript critically for important intellectual content. TH-S performed literature research and wrote the manuscript. All authors approved the final version of the manuscript.

### Conflict of interest statement

The authors declare that the research was conducted in the absence of any commercial or financial relationships that could be construed as a potential conflict of interest.
